# Developing the skills needed for successful manual small-incision cataract surgery

**Published:** 2023-12-01

**Authors:** Rengaraj Venkatesh, William Dean

**Affiliations:** 1 Chief Medical Officer: Aravind Eye Hospital; 2 Assistant Clinical Professor: ICEH, LSHTM, UK. Honorary Associate Professor: University of Cape Town, South Africa.; 3 Consultant: Speciality Director, Gloucestershire Hospitals NHS Foundation Trust, UK.

**Simulation training, preparation, and repeated practice of cataract surgical techniques will help you to become a confident and competent cataract surgeon.**

**Figure:**
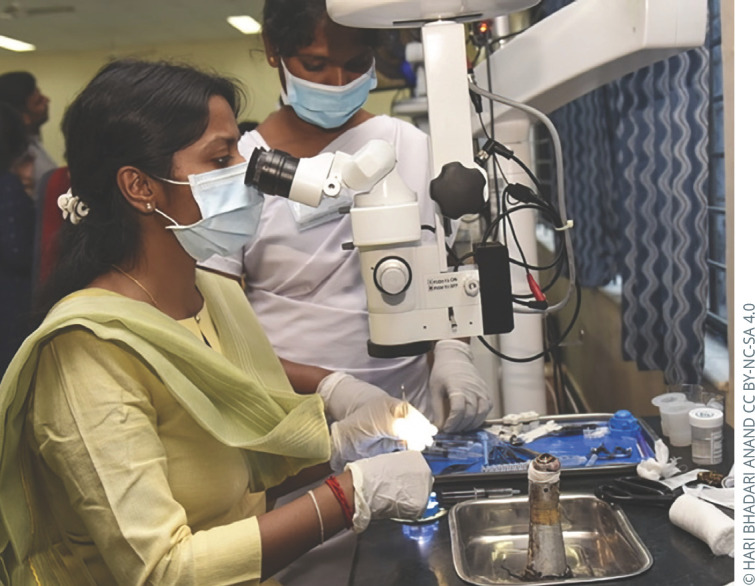
Trainees practice cataract surgical skills on a goat's eye in the wet lab. **INDIA**

Cataract simulation training is a valuable part of the journey towards surgical competence. In this article, we suggest ways to:

- Prepare for hands-on surgical training
- Use simulation to learn and practice manual small-incision cataract surgery (MSICS)
- Select patients for your first 50–100 cataract operations.

We also share some tips and clinical pearls to improve your MSICS technique.

## How to prepare for hands-on surgical training

- To ensure you are familiar with the **anatomy of the eye** and the **steps of the procedure**, study the wealth of material available in previous issues of this journal (www.cehjournal.org), online videos, and manuals of cataract surgery, and on websites such as Cybersight (www.cybersight.org).
- To develop an understanding of the **tools** of cataract surgery, practice safe handling of the equipment and surgical instruments, including the microscope.
- To better understand the **mechanics of cataract surgery**, observe senior surgeons performing cataract surgery. Pay attention to the different **approaches and surgical techniques**. Understanding the **surgical process** and **how the surgical team works**, will ensure both quality outcomes and patient safety.
- Participate in patient care to whatever degree your training allows; this will help you to develop **a professional attitude** towards patients and other eye care providers.

## Learn manual small-incision cataract surgery (MSICS) using simulation

A randomised-controlled clinical trial showed that using synthetic eyes for simulation training in manual small-incision cataract surgery (MSICS) doubled the confidence of trainees and reduced complication rates by 72% when trainees operated on patients.^1^ Similarly, a large-scale study in India showed a 52% reduction in posterior capsule rupture rates in procedures performed by trainees after they underwent skills training in the wet laboratory.^2^

A learning and teaching assessment rubric is a valuable tool for learning MSICS, and one has been specifically developed to guide practice of MSICS in simulation.^3^ Virtual reality simulators provide a three-dimensional virtual, stereoscopic surgical guide for trainees to practice cataract surgical steps.^4^ The MSICS simulators made by HelpMeSee and Orbis/FundamentalVR are discussed in two online-only articles in this issue – see www.cehjournal.org.

In a simulated environment, the basic steps of cataract surgery can be practiced individually – again and again – until the trainee is competent in that environment. The trainee should use the simulation environment to assess their own competence and confidence in each step, and – after honest reflection, or feedback from a trainer – focus their efforts where improvement is needed. Training overlays, that are available in virtual reality simulators, can be especially helpful. Displays of the depth of the dissection plane, intraocular pressure, blade angulation, adverse events, and error-indicators suggest deviations from accepted standards.

Artificial eyes are very useful for practicing scleral tunnels, corneal entry, capsulorrhexis, or capsulotomy, as well as intra-ocular lens (IOL) insertion. Apples provide an excellent and very affordable simulation medium that allows trainees to practice scleral tunnel formation using a crescent blade. Tomatoes and grapes are useful for simulating the practice of capsulorrhexis, and bovine, porcine, or goat eyes can help trainees to become familiar with the anatomy of the eye, and how it responds to interactions.

## How to select patients for your first set of cataract operations

Case selection is vital for your initial 50–100 operations. Select only low-risk patients, as this will minimise the risk of poor patient outcomes. If possible, video record the operations you perform. Reflective learning is very powerful, and it is worth using an assessment tool to evaluate your performance.^5,6^

An ideal patient for cataract surgery by a novice trainee is described below.

- Good vision in the other eye (the one not being operated on).
- Good access to the eye (known as ‘good ocular exposure’). Exclude patients with deep eye sockets, a large nasal bridge, and/or small eyes.
- Nuclear sclerosis of grades 2–3 (without posterior subcapsular cataract), with no evidence of potential zonular weakness.
- Pupillary dilation of at least 7 mm.
- A clear cornea and healthy endothelium.
- Normal axial length (22–24 mm)
- An absence of pre-existing ocular co-morbidities, including ocular trauma, glaucoma, shallow anterior chamber, pseudoexfoliation, uveitis, or posterior synechiae.
- Good general health, a mobile cervical spine, and the ability to lie still and flat for an extended period of time
- Someone whose language you, as the surgeon, can understand and speak.

## Surgical tips

1. Stabilising the globe is essential for proper tunnel construction and nucleus delivery.
2. Adequate, but light cauterisation of the scleral blood vessel makes it easier to visualise the tunnel depth during scleral groove construction.
3. Marking the scleral incision with calipers helps with the exact placement of the incision and with good tunnel construction.
4. Constructing a slightly oversized scleral tunnel, with the inner opening larger than the outer opening, reduces risk during nucleus delivery.
5. Capsular staining improves visibility and control of the capsular tear during continuous curvilinear capsulorrhexis.
6. A 7.5 mm capsulorrhexis reduces the risk of nucleus entrapment in the capsular bag. Consider a linear or envelope capsulotomy.
7. To reduce the risk of anterior chamber collapse, maintain a slight upward force on the roof of the tunnel during capsulorrhexis, hydrodissection, hydrodislocation, and cortical cleanup.
8. Respond quickly to potential capsulorrhexis runout by pulling the apex of the flap towards the centre of the tear (the Brian Little rescue technique)^7^ or by converting to a mini can-opener technique. To help prevent capsular run-out, use viscoelastic or ophthalmic viscosurgical devices or use an anterior chamber maintainer to increase pressure.
9. During hydrodissection, without pressing down on the nucleus, ensure the cannula is placed just below the capsulorrhexis margin at 3, 6, and at 9 o'clock.
10. Use ophthalmic viscosurgical devices above and below the nucleus during vectis delivery of the nucleus to protect the endothelium, iris, and posterior capsule.
11. Use the aspiration port of the Simcoe cannula to carefully engage and peel cortex off the capsular bag. This reduces stress on the zonules and enhances separation of the cortex from the capsule.
12. Ensure adequate flow of balanced saline during cortical cleanup. However, to reduce the risk of iris prolapse, avoid over-pressurising the anterior chamber.
13. Carefully control aspiration force and maintain the aspiration port in the upright position to reduce the risk of anterior chamber collapse and posterior capsular captures and tears.
14. Use an ophthalmic viscosurgical device to adequately fill the capsular bag before inserting the intraocular lens.
15. At the end of surgery, restore intraocular pressure and double check that it is adequate. A large, single air bubble after surgery is a sign that the anterior chamber is clear of viscoelastic or vitreous. Remove the bubble at the end of the operation.
